# Novel Aggregation Promoting Factor AggE Contributes to the Probiotic Properties of *Enterococcus faecium* BGGO9-28

**DOI:** 10.3389/fmicb.2017.01843

**Published:** 2017-09-26

**Authors:** Katarina Veljović, Nikola Popović, Marija Miljković, Maja Tolinački, Amarela Terzić-Vidojević, Milan Kojić

**Affiliations:** Institute of Molecular Genetics and Genetic Engineering, University of Belgrade, Belgrade, Serbia

**Keywords:** enterococci, auto-aggregation, AggE, adhesion, biofilm, probiotic

## Abstract

The understanding of mechanisms of interactions between various bacterial cell surface proteins and host receptors has become imperative for the study of the health promoting features of probiotic enterococci. This study, for the first time, describes a novel enterococcal aggregation protein, AggE, from *Enterococcus faecium* BGGO9-28, selected from a laboratory collection of enterococcal isolates with auto-aggregation phenotypes. Among them, *En. faecium* BGGO9-28 showed the strongest auto-aggregation, adhesion to components of ECM and biofilm formation. Novel aggregation promoting factor AggE, a protein of 178.1 kDa, belongs to the collagen-binding superfamily of proteins and shares similar architecture with previously discovered aggregation factors from lactic acid bacteria (LAB). Its expression in heterologous enterococcal and lactococcal hosts demonstrates that the *aggE* gene is sufficient for cell aggregation. The derivatives carrying *aggE* exhibited the ten times higher adhesion ability to collagen and fibronectin, possess about two times higher adhesion to mucin and contribute to the increase of biofilm formation, comparing to the control strains. Analysis for the presence of virulence factors (cytolysin and gelatinase production), antibiotic resistance (antibiotic susceptibility) and genes (*cylA, agg, gelE, esp, hylN, ace, efaA*^*fs*^, and *efaA*^*fm*^) showed that BGGO9-28 was sensitive to all tested antibiotics, without hemolytic or gelatinase activity. This strain does not carry any of the tested genes encoding for known virulence factors. Results showed that BGGO9-28 was resistant to low pH and high concentrations of bile salts. Also, it adhered strongly to the Caco-2 human epithelial cell line. In conclusion, the results of this study indicate that the presence of AggE protein on the cell surface in enterococci is a desirable probiotic feature.

## Introduction

Enterococci are an important group of lactic acid bacteria (LAB) which have the ability to survive various environmental conditions, allowing them to inhabit different ecological niches, such as water, soil (Giraffa, 2003), human, and animal gastrointestinal (GI) tracts (Bhardwaj et al., 2011) and food products, especially fermented dairy products (Gomes et al., 2008; Martín-Platero et al., 2009). It has been shown that enterococci have positive properties, such as enterocin production and aroma producing components (Foulquié-Moreno et al., 2006). Many enterococci isolated from fermented dairy products have proven to be great natural probiotics and are generally considered to be beneficial and safe to the host (Eaton and Gasson, 2001; Pieniz et al., 2014).

To evaluate the microorganism as novel food constituent with a health claim, several essential characteristics should be considered, including the survival throughout the GI passage, adhesion, metabolic activities and its effect on intestinal homeostasis (Miquel et al., 2015).

One of the important criteria for probiotic selection is the capability to adhere to the host's intestinal epithelium, according to Guidelines for the Evaluation of Probiotics in Food published in 2006 by the FAO/WHO working group. It is believed that adherence ability of probiotic bacteria to intestinal cells is important for successful colonization and consequently may lead to exclusion of pathogens and/or immunomodulation; expected to provide long time lasting beneficial effects for health (McNaught and MacFie, 2001; Kravtsov et al., 2008). There are also scientists who have a controversial opinion on this issue (or that it is still questionable) because proximity to the intestinal mucosa and a long transit time in the gut are not sufficient to maximize the beneficial effects of a strain (Miquel et al., 2015). Moreover, microorganisms do not have to permanently colonize the system to be achieved the effect on the host. The mechanism by which the consortium of strains from fermented milk product elicit this response is still unclear, but it seems that the effect is rapid (occurring within the first 24 h after application) and it lasts regardless of whether the consortium was introduced during a 1 day period, or with subsequent repeated applications over a several weeks (McNulty et al., 2011).

The precise mechanisms of host-microbe interaction remain still unclear, although there is growing evidence that adherence to the components of the extracellular matrix (ECM) such as mucin, fibronectin, fibrinogen, collagen or laminin (Lorca et al., 2002; Yadav et al., 2013) depends on bacterial cell-surface composition. The expression of ECM-binding proteins on the surface of pathogenic bacteria provide adherence to distinct components of the ECM. The presence of enterococcal surface proteins has been shown to increase the persistence of bacteria in the urinary bladders of mice (Shankar et al., 2001). Collagen binding protein from *Enterococcus faecalis* has been shown to be a putative virulence factor involved in the colonization of renal tissue (Lebreton et al., 2009). On the other hand, probiotic microorganisms express cell-surface adhesins that mediate microbial adhesion to ECM components of host tissue. The expression of aggregation factors on the cell surface of bacteria could induce cell aggregation, visible as auto-aggregation, an important property for colonization of the oral cavity, human gut or urogenital tract.

In LAB and bifidobacteria strains, the ability of adhesion and auto-aggregation has been reported to be significantly related (Del Re et al., 2000). Correlations between the adhesion, aggregation and surface charges were observed among the *Lactobacillus fermentum* and *Lb. paracasei* strains (Piwat et al., 2015). Aggregation promoting factors from LAB differ in molecular weight and primary structure. The best characterized aggregation factors of high molecular weight (proteins of molecular mass >170 kDa), which are responsible for forming large cell aggregates, causing strong auto-aggregation and directly involved in adhesion to collagen and fibronectin, are from *Lactococcus lactis* subsp. *lactis* BGKP1 (Kojic et al., 2011), *Lb. paracasei* subsp. *paracasei* BGNJ1-64 (Miljkovic et al., 2015) and *Lb. paracasei* subsp. *paracasei* BGSJ2-8 (Lozo et al., 2007).

This paper, for the first time, describes the occurrence of novel enterococcal aggregation protein AggE from *En. faecium* BGGO9-28, selected from a laboratory collection as a strain, which belongs to a group with strong aggregation ability. The objectives of this study were to assess the adhesion and aggregation abilities of *En. faecium* BGGO9-28 and to determine a possible correlation between the adhesion of cells and aggregation ability. The novel plasmid-located *aggE* gene was cloned, sequenced and expressed in homologous and heterologous enterococcal and lactococcal hosts, showing that AggE protein is sufficient for cell aggregation in all tested hosts. AggE aggregation factor, protein of 178.1 kDa shares the highest identity with AggL protein from lactococci (81.4%). In addition to its adherence property, BGGO9-28 fulfills several essential criteria required to be considered as a potential probiotic strain: absence of genes coding for virulence factors, the ability to survive in the presence of gastric juices, bile salts and intestinal juices, and strong adhesion ability to host intestinal cells.

## Materials and methods

### Bacterial strains, media, and growth conditions

The *Enterococcus* and *Lactococcus* strains used in this study (Table 1) were grown in M17 broth (Merck, GmbH, Darmstadt, Germany) supplemented with glucose (0.5% w/v) (GM17) at 30°C. Enterococcal and lactococcal transformants were grown in GM17 medium supplemented with erythromycin (10 μg/ml). *Escherichia coli* DH5α was grown in Luria-Bertani broth (LB) at 37°C aerobically. Solid medium plates were prepared by adding 1.7% agar (Torlak, Belgrade, Serbia) into each medium broth.

**Table 1:** Bacterial strains and plasmids used in this study.

**Strains**	**Source**	**Strain characteristics**	**Source or references**
***Enterococcus*** **sp**.			
BGGO9-27	Semi-fat 30 days artisanal old cheese	Agg^+^	Terzic-Vidojevic et al., 2014
BGGO9-28		Agg^+^	
BGGO9-30		Agg^+^	
BGGO9-32		Agg^+^	
BGGO9-33		Agg^+^	
BGGO9-56		Agg^+^	
BGGO10-37		Agg^+^	
BGGO10-38		Agg^+^	
BGGO11-27	Semi-fat 10 days artisanal old cheese	Agg^+^	Golić et al., 2013
BGGO11-29		Agg^+^	
BGGO11-33		Agg^+^	
BGGO11-41		Agg^+^	
BGDU4-1	Semi-had, semi-fat, 15 days old cheese	Agg^+^	Laboratory collection
BGDB23-11	Semi-had, full-fat, 12 days old cheese	Agg^+^	Laboratory collection
BGZLS10-27	Semi-fat 10 days artisanal old cheese	Agg^−^	Valenzuela et al., 2009
BGZLS10-27/pAggES10	Derivative of BGZLS10-27 carrying pAggES10	Agg^+^, Em^r^	This study
BGZLS10-27/pAggEPX48	Derivative of BGZLS10-27 carrying pAggEPX48	Agg^+^, Em^r^	This study
***Lactoccoccus lactis*** **subsp**. ***lactis***			
BGKP1		Agg^+^	Kojic et al., 2011
***Lactococcus lactis*** **subsp**. ***cremoris***			
MG7284	Derivative of MG1363	Fus^r^, Spc^r^, Agg^−^	Gasson, 1983
MG7284/pAggES10	Derivative of MG7284 carrying pAggES10	Agg^+^, Em^r^	This study
MG7284/pAggEPX48	Derivative of MG7284 carrying pAggEPX48	Agg^+^, Em^r^	This study
***Escherichia coli***			
DH5α		*supE44 ΔlacU169 (ø80 lacZΔM15) hsdR17 recA1 endA1 gyrA96 thi-1 relA1*	Hanahan, 1983
**PLASMIDS AND CONSTRUCTS**			
pAZIL	Shuttle cloning vector	Em^r^	Kojic et al., 2011
pAggES10	Derivative of pAZIL vector	Carrying *Stu*I fragment of 8,944 bp from BGGO9-28	This study
pAggEPX48	Derivative of pAZIL vector	Carrying *Pvu*I-*Xba*I fragment of 7,115 bp subcloned from pAggES10	This study

*Agg^+^, aggregation-positive; Agg^−^, aggregation-negative; Em^r^, resistance to erythromycin; Fus^r^, resistance to fusaric acid; Spc^r^, resistance to spectinomycin*.

### Identification of strains

Primary identification of enterococcal isolates was done by catalase testing, Gram staining and cell morphology, hydrolysis of arginine, CO_2_ production from glucose and formation of black zones on bile esculin agar (Himedia, Mumbai, India). Sequencing of genes for 16S rRNA of BGGO9-28 was performed using primers: UNI16SF (5′-GAGAGTTTGATCCTGGC-3′) and UNI16SR (5′-AGGAGGTGATCCAGCCG-3′) (Jovcic et al., 2009).

The Polymerase Chain Reaction (PCR) amplicons were purified using a GeneJet PCR Purification Kit (Thermo Scientific, Lithuania) and sequenced (Macrogen Europe, Amsterdam, The Netherlands). The Basic Local Alignment Search Tool (BLAST) algorithm was used to determine the most related sequences in the NCBI nucleotide sequence database (http://www.ncbi.nlm.nih.gov/BLAST). Based on 16S rRNA gene sequencing results the BGGO9-28 strain was classified as *En. faecium* (submitted to the European Nucleotide Archive (ENA) under accession No: LT222049).

### Auto-aggregation assay

The auto-aggregation ability of the selected enterococci strains was tested according to the method of García-Cayuela et al. (2014). Percentage of auto-aggregation was determined using the equation: [1 − (At/A0) × 100] where At represents the absorbance at different time points (1, 2, 3, 4, and 5 h) and A0 is absorbance at time 0 of three independent measurements.

### *In Vitro* adhesion ability to components of ECM

The collagen binding ability of all the selected enterococci strains and derivates was tested according to Miljkovic et al. (2015). The wells were coated with 100 μg/ml of type I collagen from rat tail (BD Bioscience, New Jersey, USA). The ability of the tested strains to bind to fibronectin was assessed as previously described by Ahmed et al. (2001). The wells were coated with 100 μg/ml human fibronectin (Serva, Heidelberg, Germany). The ability of the tested strains to bind to mucin was tested according to Muñoz-Provencio et al. (2009) with modification. The wells were coated with 100 μg/ml mucin from porcine stomach (Sigma, St. Louis, USA). After coating with collagen/fibronectin/mucin, wells were washed with PBS and blocked with bovine serine albumin (BSA) (2% in PBS). Also, empty wells were coated with BSA (control wells). Upon removal of BSA solution and washing of wells with PBS, the test cultures (100 μl, 10^8^ CFU/ml) were added and plates were incubated on an orbital platform shaker for 2 h at 37°C. Non-adhered cells were removed by washing of the wells three times with 200 μl of PBS. The adhered cells were fixed at 60°C for 20 min and stained with crystal violet (100 μl/well, 0.1% solution) for 45 min. Wells were subsequently washed tree times with PBS to remove the excess stain. The stain bounded to the cells was dissolved by 100 μl of citrate buffer (pH 4.3) and absorbance was measured at 570 nm. The results are presented as the average of absorbance values per each strain (normalized with respect to absorbance values of BSA-coated wells) from three independent experiments per strain.

### Biofilm formation assay

The ability of the enterococci to form biofilm was assessed as previously described by Peter et al. (2013) with minor modifications. The culture (10^8^ CFU/mL) was diluted to 1:40 in GM17 and 200 μl of dilution was used to inoculate sterile 96 well-polystyrene microtiter plates. After incubating for 24 h at 37°C, the excess bacterial cells were removed (the removal of “planktonic bacteria”) and wells were gently washed tree times with PBS, dried in an inverted position and stained with crystal violet (100 μl/well, 0.1% solution) for 45 min. The wells were rinsed again with PBS and retained crystal violet was solubilized in 200 μl of ethanol - acetone (80:20) mixture. The absorbance was measured at 570 nm. The results were presented as average of absorbance values per each strain (normalized with respect to absorbance values of empty wells) from three independent experiments.

### Southern blot hybridization

Transfer of DNA from agarose gel to membrane, labeling of probe and detection of hybrids was performed as recommended by the manufacturer of the DIG DNA Labeling and Detection Kit (Roche, Mannheim, Germany). Hybridizations were carried out at 65°C.

### Cloning and expression of novel enterococcal aggregation promoting factor AggE

The procedure described by O'Sullivan and Klaenhammer (1993) was applied for plasmid isolation from enterococci and lactococci.

For preparation of plasmid libraries, plasmid DNA from *En. faecium* BGGO9-28 was digested separately with *Xba*I and *Stu*I restriction enzymes. The resulting DNA fragments were cloned into the pAZIL vector and plasmid libraries were screened in *E. coli* DH5α. All derivatives carrying different fragments were transferred into non-aggregating derivatives *Lc. lactis* subsp. *cremoris* MG7284 (Agg^−^) and *En. faecalis* BGZLS10-27 (Agg^−^).

All relevant constructs were sequenced (Macrogen, Amsterdam, The Netherlands). The BLAST program was used for sequence annotation and analysis of sequence similarities. ORF prediction was obtained by DNA Strider. Motif Scan (http://myhits.isb-sib.ch/cgi-bin/motif_scan), and Superfamily 1.75 (http://supfam.org/) programs were used to analyse AggE protein.

Comparative analyses of enterococcal (AggE) and lactococcal (AggL) aggregation factors were performed by PCR reactions. The primers used in PCR amplification were: AggE-1DF (5′-GACTGCTAAGTCAACGGGGG-3′) and AggE-1DR (5′-GATAGGTAATATTTGCTGG-3′). Total DNA (1 ng) was mixed with 17.75 μl of bidistilled water, 2.5 μl of 10 × PCR buffer (Fermentas, Lithuania), 1 μl dNTP mix (10 mM), 1.5 μl of MgCl_2_ (25 mM), 1 μl (10 pmole) of each primer and 0.25 μl of Taq polymerase (Fermentas, Lithuania). Performed using the GeneAmp 2700 PCR Cycler (Applied Biosystems, Foster City, California, USA), the PCR program consisted of 5 min at 96°C, 30 cycles of 96°C for 30 s, 40°C for 30 s and 72°C for 60 s, and an additional extension step of 5 min at 72°C.

### Haemolytic and gelatinase activities

Haemolytic activity of enterococcal strain BGGO9-28 was tested on Columbia Blood Agar (Oxoid LTD, Basingstoke, Hampshire, England) containing 5% (v/v) defibrinated horse blood after 48 h of incubation at 37°C. Production of β-haemolysin was detected as the appearance of clear zones around colonies. Also, production of gelatinase of enterococcal strain BGGO9-28 was tested on agar plates containing 30 g of gelatine (Difco, Detroit, MI, USA) per liter as described by Lopes et al. (2006). After filling the Petri plate with 550 g/l ammonium sulfate, the appearance of clean zones around colonies indicated gelatinase production.

### Susceptibility testing

The antibiotic susceptibility of enterococcal strain BGGO9-28 was done by determining the minimum inhibitory concentrations (MICs) by microdilution testing following The Clinical and Laboratory Standards Institute (CLSI), 2015 criteria. Susceptibility was tested against: ampicillin (4–16 μg/ml), vancomycin (4–32 μg/ml), erythromycin (0.5–8 μg/ml), tetracycline (4–16 μg/ml), ciprofloxacin (1–4 μg/ml), nitrofurantoin (32–128 μg/ml), chloramphenicol (8–32 μg/ml), linezolid (2–8 μg/ml) and gentamicin (>500 μg/ml). After 24 h incubation at 30°C, cell density was determined spectrophotometrically by measuring absorbance at 595 nm using a Plate Reader Infinite 200 pro (MTX Lab Systems, Austria). MIC values were determined as the lowest concentration of antibiotic that inhibits visible growth of bacteria.

### PCR detection of virulence determinants

In order to test for the presence of genes which indicate virulence, determinants cytolysin (*cylA*), aggregation factor (*agg*), gelatinase (*gelE*), enterococcal surface protein (*esp*), cell wall adhesions (*efaA*^*fs*^, and *efaA*^*fm*^), collagen adhesin (*ace*), and hyaluronidase (*hylN*) (Eaton and Gasson, 2001; Vankerckhoven et al., 2004) the total DNA of *En. faecium* BGGO9-28 was used.

### Survival in simulated GI tract

Survival of enterococcal strain BGGO9-28 in a simulated GI tract was performed according to Nikolic et al. (2012). Strain was grown in GM17 for 24 h and culture was harvested by centrifugation (10,000 g for 10 min), washed twice with 0.85% NaCl and concentrated 10-times in reconstituted (10%) sterile skimmed-milk (Difco, Becton Dickinson, Franklin Lakes, NJ, USA). Afterwards, bacterial suspensions were diluted 10-times with gastric juice (125 mM NaCl, 7 mM KCl, 45 mM NaHCO3, 0.3% pepsin (Sigma, St. Louis, MO, USA) adjusted to pH 2.0 with HCl), incubated for 90 min at 37°C in aerobic conditions under shaking. Then, bacterial suspensions were centrifuged (2,050 g, 15 min), resuspended in duodenal juice [1% bovine bile (Sigma, St. Louis, MO, USA) adjusted with 10 M NaOH to pH 8.0] and incubated for 10 min at 37°C in anaerobic conditions. Finally, harvested cell suspensions were resuspended in intestinal juice (0.3% bovine bile, 0.1% pancreas acetone powder porcine type I (Sigma, St. Louis, MO, USA), pH 8.0) and incubated for 120 min at 37°C in anaerobic conditions. Determination of viable counts was carried out in the initial cultures and after each of the challenges (gastric, bile salt, and intestinal juices). Serial dilutions of the samples were made and inoculated on the surface of GM17 agar using spread plate technique. Viable colony counts were determined and survival rate was presented as means of log units of BGGO9-28 growth. Experiments were carried out in triplicate.

### Adhesion to Caco-2 cell lines

The colonocyte-like cell line Caco-2, was used to determine the adhesion ability of enterococcal strain BGGO9-28. A Caco-2 cell line was purchased from the European Collection of Cell Cultures (ECACC No. 86010202). Adhesion to the Caco-2 cell line was done according to Sánchez et al. (2010). The results of adhesion were expressed as percentage (%) (colony forming units (CFU) adhered bacteria/CFU added bacteria × 100) from two replicated plates. In each plate three wells were used per sample.

### Statistical analysis

Differences between treatments were examined for significance by a Student's *t*-test after analysis of variance (Kirkman, 1996).

## Results and discussion

The important goals of this study were to investigate the aggregation ability of *Enterococcus* sp. in general, and to examine the possible role of *Enterococcus* aggregation factor as a potential probiotic feature.

### Identification and auto-aggregation of selected enterococci

Considering the importance of aggregation phenomena for human health, the laboratory collection of enterococci was screened for strains exhibiting strong aggregation ability for further analysis. Fourteen *Enterococcus* strains were selected from among 636 examined enterococcal isolates (Terzić-Vidojević et al., 2015) from a laboratory collection, based on their ability to cause auto-aggregation of the cells, using an aggregation visual assay. All of them were isolated from artisanal homemade cow's milk cheeses, produced in households of the Golija and Valjevo mountain regions of the Republic of Serbia (Table 1). All enterococcal isolates were Gram positive and catalase negative cocci, in pairs or short chains. Hydrolysis of arginine and formation of black zones on bile esculin agar were positive. None of the enterococci chosen were able to produce CO_2_ from glucose. It was found that aggregation ability is a rare phenotype among enterococci (2.2% in our laboratory collection) similarly as in other LAB (Miljkovic et al., 2015).

Some LAB, including enterococcal species, represent beneficial microbiota existing in the human and animal GI and urogenital tracts (Bhardwaj et al., 2011). One beneficial property of bacteria with auto-aggregation ability is the prevention of colonization of pathogenic bacteria by the formation of a barrier via auto-aggregation to intestinal mucosa (O'Toole and Cooney, 2008; Prince et al., 2012).

**Figure 1:**
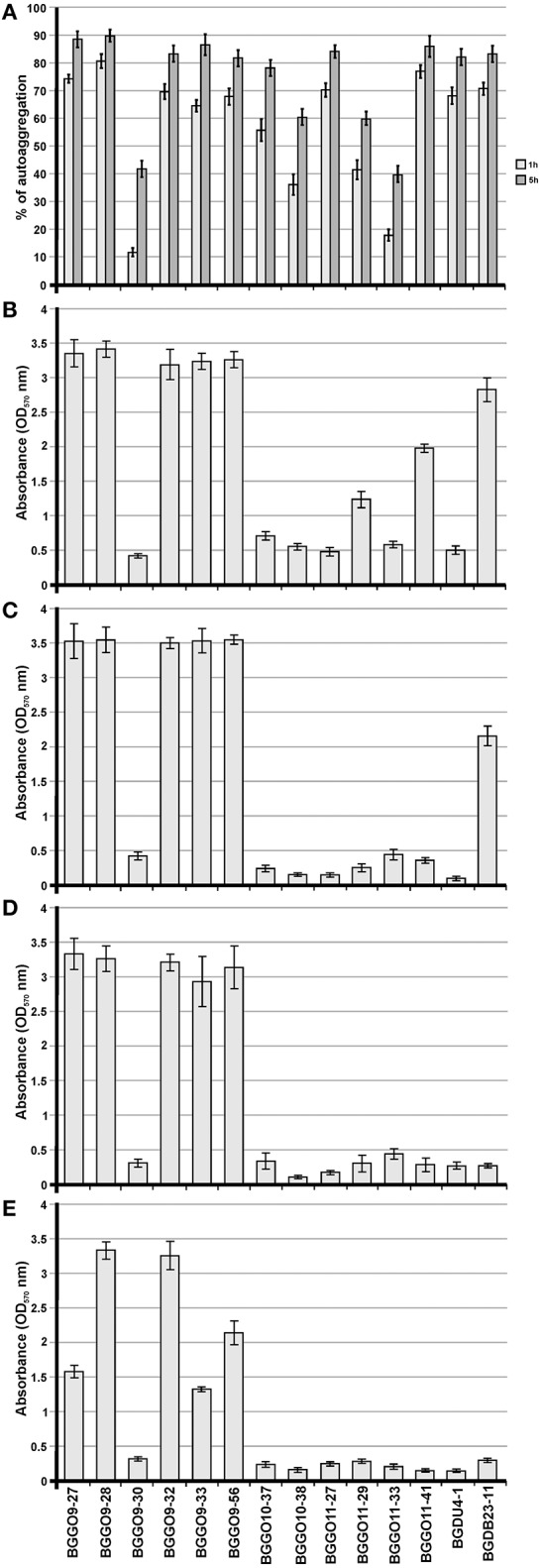
Adhesion capability of selected enterococal strains. Graphical presentation of results obtained in **(A)** auto-aggregation assay (auto-aggregation ability is expressed as percentages); **(B)** collagen-binding assay; **(C)** fibronectin-binding assay; **(D)** mucin-binding assay; **(E)** biofilm formation assay of selected strains (results were expressed as average of normalized A_570_ values). The error bars show the standard deviations.

Based on a visual assay, auto-aggregation ability was further confirmed by monitoring the changes in absorbance (OD_600_) of cultures during 5 h of sedimentation at 30°C. All analyzed enterococci strains showed varying levels (1 h: 5.34%–81.09%; 5 h: 29.825–89.86%) of auto-aggregation ability and can be classified in three groups: (*i*) strong auto-aggregation (strains BGGO9-27, BGGO9-28, BGGO9-32, BGGO9-33, BGGO9-56, BGGO10-37, BGGO11-27, BGGO11-41, BGDU4-1, and BGDB23-11; with more than 80% of precipitated cells for 5 h); (*ii*) medium auto-aggregation (strains BGGO10-38 and BGGO11-29; with about 60% of the precipitated cells for 5 h); and (*iii*) low auto-aggregation (strains BGGO9-30 and BGGO11-33; with about 40% of the precipitated cells for 5 h) (Figure 1A).

### Adhesion of selected enterococci to components of ECM

One of the important goals of this study was to investigate the possible role of *Enterococcus* aggregation factor in its adherence to the main components of ECM, such as collagen (Figure 1B), structural glycoprotein fibronectin (Figure 1C) and mucin (Figure 1D), in order to assess its probiotic potential. The strains most adhesive to all tested components of ECM were BGGO9-27, BGGO9-28, BGGO9-32, BGGO9-33, and BGGO9-56. The rest of the strains showed a difference in affinity for various components of ECM. Interestingly, strains BGGO11-29 and BGGO11-41 exhibited the ability to bind collagen (absorbance value 1.17 and 1.97, respectively), while binding to fibronectin and mucin was absent (absorbance value is less that 0.4). Also, it was noted that strain BGDB23-11 binds to collagen and fibronectin (absorbance value 2.82 to collagen and 2.16 to fibronectin), but binding to mucin was very poor (absorbance value 0.28).

The results obtained for auto-aggregation and adhesion to components of ECM among the selected enterococcal strains indicate that they possess a different kind of molecules present on their surface that are involved in cell-to-cell interaction. However, the selected enterococci can be classified into at least four groups: group I (consisting of strains BGGO9-27, BGGO9-28, BGGO9-32, BGGO9-33, and BGGO9-56) showed strong ability to bind to collagen, fibronectin and mucin, while at the same time exhibiting very powerful auto-aggregation ability; group II (consisting of strains BGGO9-30, BGGO10-37, BGGO10-38, BGGO11-27, BGGO11-33, and BGDU4-1) showed a moderate to high auto-aggregation ability, but poor adhesion to all ECM components; group III (consisting of strains BGGO11-29 and BGGO11-41) showed a moderate to high auto-aggregation ability and binding to collagen, but poor adhesion to other ECM components; and group IV (consisting of strain BGDB23-11) showed high auto-aggregation ability and binding to collagen and fibronectin, but poor adhesion to mucin.

### Biofilm formation of selected enterococci

Excluding many environmental factors, the number of genetic factors known to be involved in biofilm production has increased in recent years (Mohamed and Huang, 2007). Different surface molecules, such as polysaccharides, have been implicated in biofilm formation. An *En. faecalis* gene mutation which encodes a putative glycosyltransferase that is involved in polysaccharide synthesis showed a 73% reduction in biofilm formation (Xu et al., 2000). In this context, we evaluated biofilm formation by the selected enterococcal strains and investigated the possible correlation between the presence of aggregation factor on the cell surface and the ability to form biofilm.

Cells of the tested strains showed differing abilities to form biofilm. Among the tested enterococci, strains BGGO9-28 and BGGO9-32 showed the strongest ability to form biofilms on the plates (plastic surface), and three strains (BGGO9-27, BGGO9-33, and BGGO9-56) showed moderate biofilm formation ability (Figure 1E). Two strains, BGGO9-28 and BGGO9-32, belong to the aforementioned group I of strains (among the tested enterococci), which also showed strong auto-aggregation ability and adhesion to components of ECM. This is the first study that indicates the existence of a relationship between biofilm formation and aggregation in non-pathogenic enterococcal strains.

### Localization of *aggE* gene responsible for auto-aggregation phenotype

**Figure 2:**
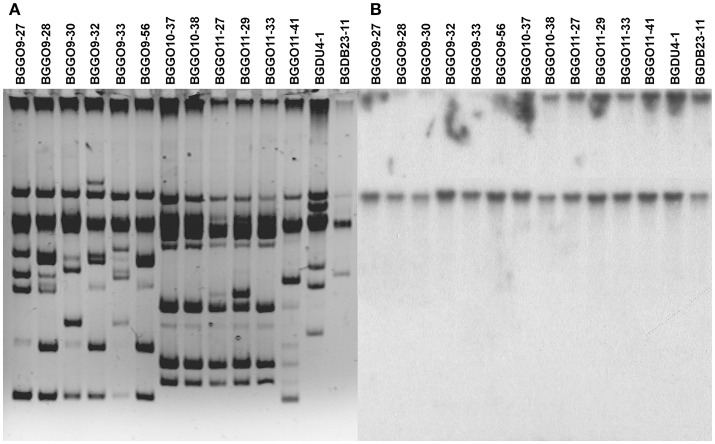
Localization of genes correlated with auto-aggregation ability. **(A)** The electrophoresis of total plasmid DNA of selected enterococcal strains expressing aggregation ability from laboratory collection on 1% agaroze gel; **(B)** Southern blot hybridization with *aggL* gene probe.

The autochthonous plasmids of the selected enterococcal strains were analyzed to confirm the location of genes correlated with auto-aggregation ability (Figure 2). To determine the relationship between the presence of plasmids and the auto-aggregation phenotype, which was very similar to that obtained for lactococci and lactobacilli (Kojic et al., 2011; Miljkovic et al., 2015), Southern blot hybridizations with *aggL* and *aggLb* gene probes from *Lc. lactis* BGKP1 and *Lb. paracasei* subsp. *paracasei* BGNJ1-64 were performed (separately) using plasmid DNA isolated from all selected enterococci. The results indicate that the gene(s) similar to *aggL* determining auto-aggregation in all selected enterococcal strains are plasmid located (on the largest common plasmid; Figure 2), which allows fast horizontal transfer among bacteria of the same niche and most probably a selective advantage for carriers. It is interesting that in enterococci, like in lacococci and lactobacilli, genes encoding for this type of aggregation factor are located on plasmids of different size indicating possible common plasmid origin. In selected enterococci it seems that plasmids carrying *aggE* gene are almost the same size even aggregation positive strains were isolated from different locations. According to plasmid profiles of selected strains it is possible to noted four groups of strains: two big groups (consisted of seven and five strains) with many common plasmids and two groups each consisted of one strain. Unfortunately, it is not possible to give final conclusion regarding plasmids carrying *aggE* gene since all analyzed strains belong to the same collection isolated from narrow distance; additional data from other groups are necessary. During propagation of selected enterococcal strains no loss of aggregation phenotype was noticed, indicating that plasmids are stable (structurally and segregationally) without any selection indicating that it could contributes to the fitness of carrier strains.

### Cloning and heterologous expression of AggE aggregation factor from strain BGGO9-28

Since the strain BGGO9-28 showed the strongest adhesion capability among selected enterococal strains, it was chosen for the cloning of *aggE* by constructing a plasmid library in pAZIL vector. All plasmid library constructs, carrying different fragments of the total plasmids of strain BGGO9-28, amplified in *E. coli*, were transferred into non-aggregating heterologous plasmid-free lactococcal strain MG7284 and enterococcal strain BGZLS10-27. Only the construct pAggES10 (carrying a *Stu*I fragment of 8,944 bp) provided auto-aggregation ability to strains MG7284 and BGZLS10-27. After the auto-aggregation capacity of construct pAggES10 was confirmed, the cloned fragment was completely sequenced. On the fragment was found an ORF with 70.6% identity with *aggL* from *Lc. lactis* BGKP1 (Kojic et al., 2011), responsible for aggregation in lactococci, and was named *aggE*. In addition, an upstream promoter region of *aggE* showed very high identity (96%) with the promoter region of *aggL* gene from lactococci. The DNA sequence of the region carrying the *aggE* from strain BGGO9-28 was submitted to the European Nucleotide Archive under accession No: LT222050.

**Figure 3:**
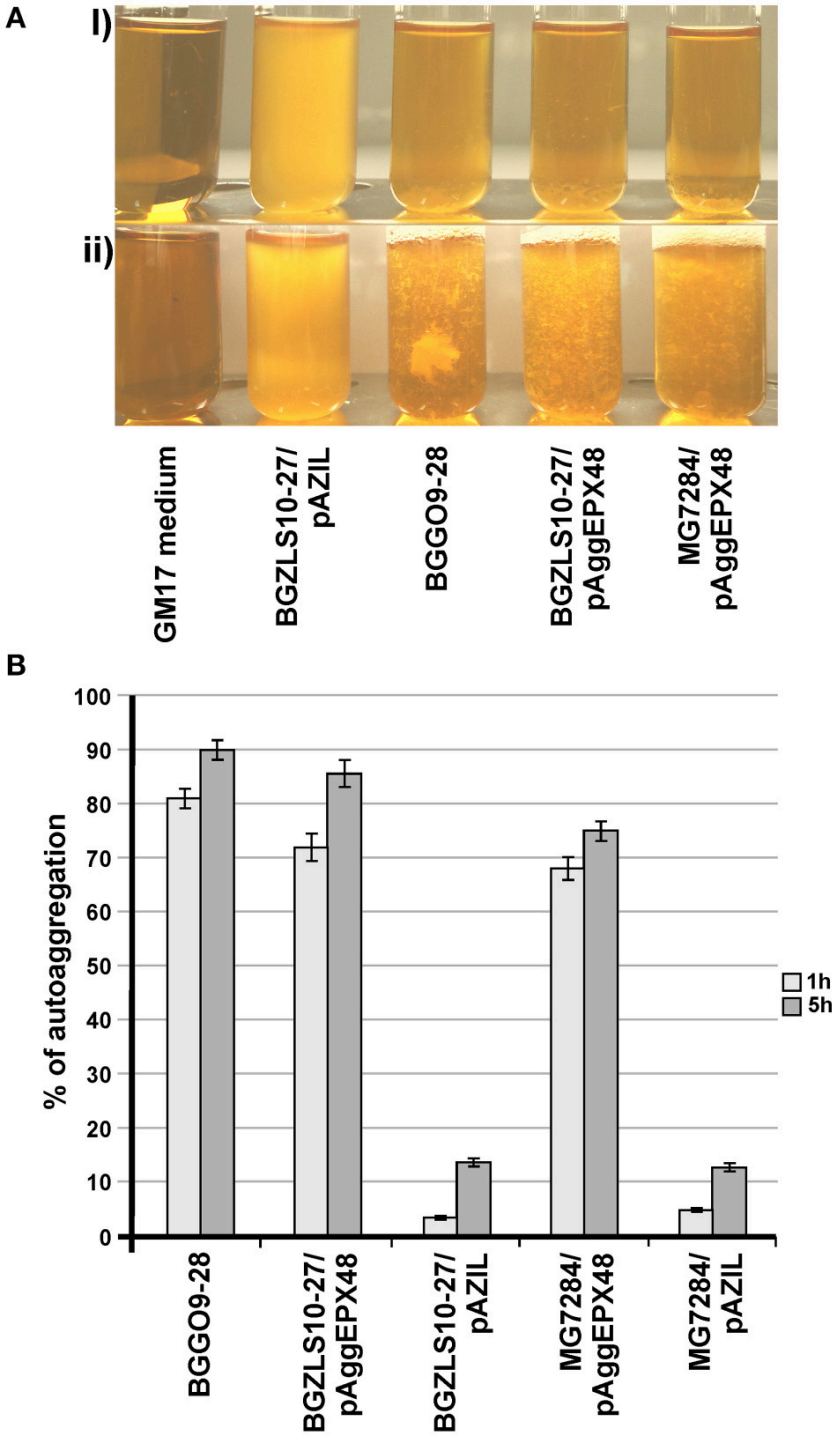
Heterologous expression of AggE aggregation factor from BGGO9-28. Aggregation ability of *En. faecium* BGGO9-28 and transformants (carryng construct pAggEPX48) in growth medium after **(A-i)** overnight cultivation and **(A-ii)** vigorous mixing; **(B)** graphical presentation of results obtained in auto-aggregation assay; auto-aggregation ability is expressed as percentages; the error bars show the standard deviations of three independent observations.

Based on the sequence analysis, a shortened *Pvu*I/*Xba*I fragment of 7,115 bp that contained only the *aggE* was subcloned into the pAZIL vector. The resulting construct, named pAggEPX48, successfully restored aggregation phenotype in strains MG7284 and BGZLS10-27, after transformation (Figure 3). It was noted that *aggE* is sufficient for the expression of aggregation phenotype in closely related LAB species (lactococci and enterococci). The result supports the conclusion that aggregation ability was stronger in transformants of enterococcal strain BGZLS10-27 compared to that of lactococcal MG7284, in contrast to results obtained with lactococcal *aggL* indicating that there are structural adaptations specific to each group of bacteria. It was found that *aggE* is surrounded by two integrase genes; upstream of *aggE* gene are located genes for RepA protein and for an integrase with high identity with the same genes on an unnamed plasmid from *En. faecium* strain UW8175, and downstream are located genes for integrase, magnesium and cobalt transport protein CorA, and a gene for protein OrfX. A constellation of genes on the sequenced fragment (RepA and OrfX separated by a cassette) and the presence of the genes for integrase on both sides of *aggE* gene indicate a possible horizontal gene transfer.

### *In Silico* analysis of AggE protein

**Figure 4:**
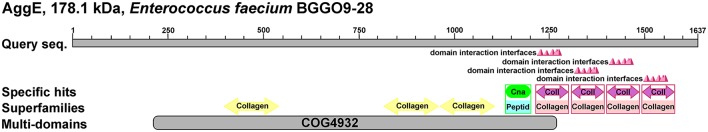
Schematic representation of AggE protein, 178.1 kDa *Enterococcus faecium* BGGO9-28. Prediction of putative conserved domains in AggE protein was analyzed by protein BLASTP search. COG4932-Uncharacterized surface anchored protein, Collagen, Collagen binding domain; Peptid, peptidase associated domain; Coll, repeat unit of collagen binding protein domain B; Cna, Cna protein B-type domain.

Primary structural analysis of AggE protein revealed that it is a 178 kDa (1,637 amino acids) membrane-anchored protein rich with amino acids threonine (12.6%) and lysine (9.3%). A BLAST search showed that AggE shares the highest identity with AggL from lactococci (81.4%) and a recently discovered hypothetical protein from *En. faecium* (WP_058138517.1) (69.6%), which is shorter by 386 amino acids. It was shown that AggE contains several motifs in relation to cell adhesion, like collagen-binding (three heterologous domains with less than 25% identity at positions 411–534, 829–944, and 964–1,089), collagen-binding B domains (four almost identical domains with consensus sequence TSVSGQKTWSDHDNQDGVRPDEITVNLLADGKKVDSKTVTAKDGWKYEFNDLDKFKGQEIKYTVAEAAVDGYKTTYDGNNIVNTH at positions 1,214–1,299, 1,304–1,389, 1,394–1,479, and 1,484–1,567), a CnaB-like domain (at position 1,132–1,200) as well as a cell wall anchoring domain (LPXTG) at the C-terminus (Figure 4).

**Figure 5:**
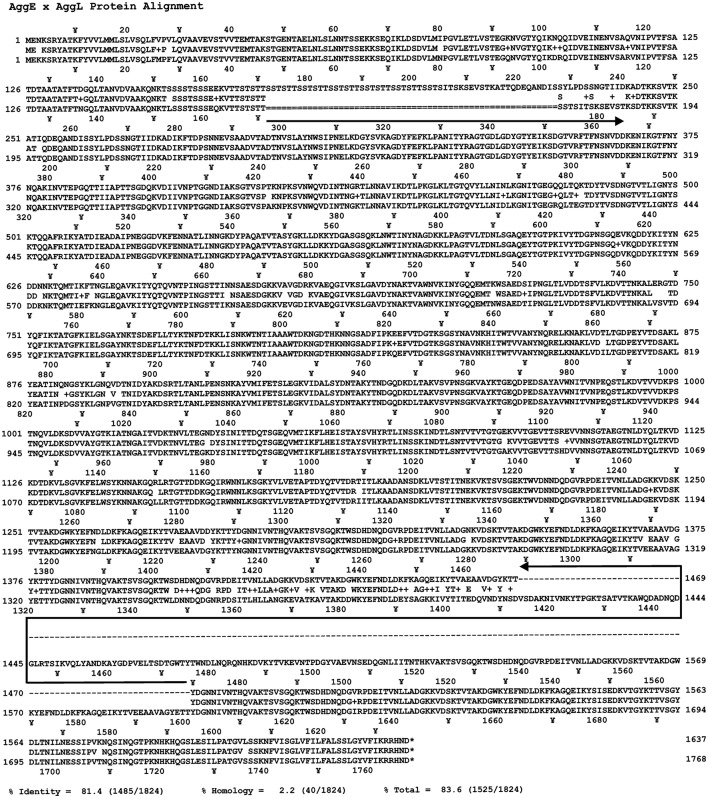
Comparison of the amino acid sequence of AggL and AggE aggregation factors using Strider software. Black arrow oriented to the right indicates the absence of amino acids in AggL protein corresponding to the region between amino acids 171 and 228 of AggE. Black arrow oriented to the left indicates the absence of amino acids in AggE protein corresponding to the region between amino acids 1,413 and 1,600 of AggL. +, − Indicates for amino acids belonging to the same physicochemical group.

**Figure 6:**
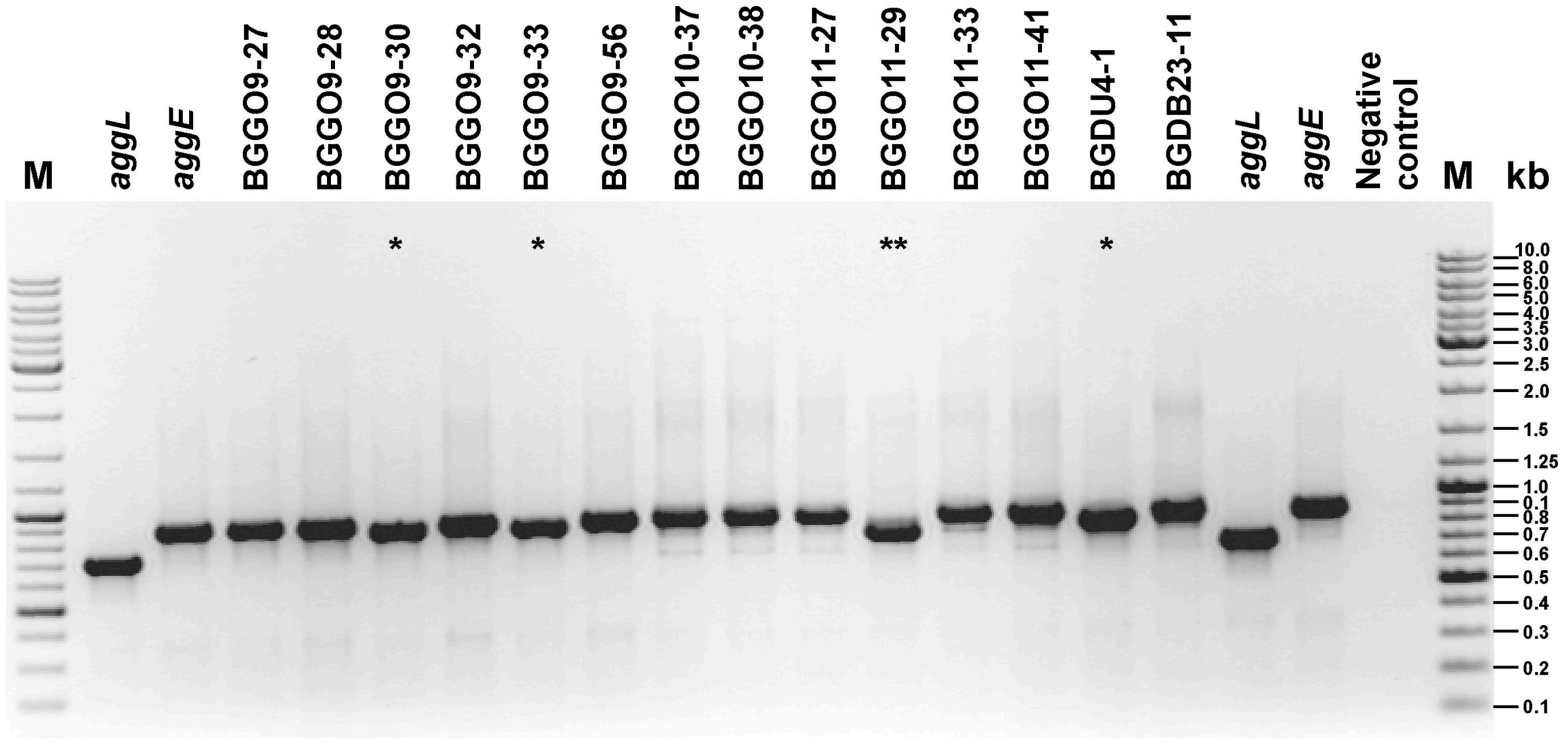
Comparation of *aggE* and *aggL* genes by PCR analyses. The electrophoresis of PCR fragments amplified using primers AggE-1DF and AggE-1DR on plasmid DNA of enterococcal strains and clones (*aggE* and *aggL*) on 1% agarose gel. ^*^Little shorter fragment than obtained for *aggE*; ^**^Fragment thereof according to the size of between *aggE* and *aggL*; M, GeneRuler DNA Ladder Mix (Thermo Scientific); kb, kilobase.

Aggregation substance (AS) of *En. faecalis*, a sex pheromone plasmid encoded cell surface proteins (Asp1, Asa1 and Asc10; these proteins share more than 90% identity throughout of the protein), mediates the formation of bacterial aggregates, thereby promoting plasmid transfer. The aggregation ability encoded by *asp1* gene was first characterized as a virulence factor of 142 kDa (Galli et al., 1990). It was proposed that amino acid motifs Arg-Gly-Asp-Ser (RGDS) and Arg-Gly-Asp-Val (RGDV) in that aggregation protein play a crucial role in adherence to eukaryotic cells. In this study we have discovered a novel aggregation promoting factor, AggE, responsible for strong aggregation of enterococcal cells. Both types of the enterococcal aggregation proteins (AS and AggE) are high molecular mass proteins rich by amino acids threonine and lysine. The difference between them is that novel aggregation factor AggE from enterococci shows different architecture compared to that characterized as a virulence factor and does not contain RGDS or RGDV amino acid motifs. In addition these two types of aggregation proteins exhibit different binding affinity to ECM proteins: the presence of AS increased enterococcal adherence to fibronectin more than eight-fold and to type I collagen more than two-fold (Rozdzinski et al., 2001) while AggE protein provides an almost identical affinity for both the collagen and the fibronectin, which has been increased more than 10 times. AggE enterococcal aggregation promoting factor showed a structure similar to aggregation factors discovered in other LAB composed of collagen binding and CnaB-like domains (Kojic et al., 2011; Miljkovic et al., 2015). The highest level of identity, 81.4%, was detected with an aggregation protein of lactococci (AggL). Comparative analysis of these two proteins indicated their common origin. Differences between these two proteins were seen only in two regions: a region between amino acids 171 and 228 of AggE (that region is deleted in AggL since it is composed of multiple repeated SSTSTT sequences), and a second region between amino acids 1,400 and 1,600 of AggL that is not present in AggE and contains two collagen binding B domains (Figure 5). These two differences may be the result of an independent selection in each organism and are most likely responsible for the different expression of aggregation proteins in lactococci and enterococci (AggL is better expressed in *Lactococcus* while AggE is better expressed in *Enterococcus*). The hypothesis of a common origin of these two genes is supported by two additional facts; an almost identical promoter region and the presence of the integrase genes close to *agg* gene. In order to determine whether the differences in *aggE* (from BGGO9-28) are a feature of all Agg^+^ enterococci, the region (which surrounds the first deletion in *aggL*, positioned between nucleotides 511–680 of *aggE*) was amplified using primers AggE-1DF (positioned in both, *aggL* and *aggE* genes between nucleotides 129–148) and AggE-1DR (positioned in *aggL* between nucleotides 817–835 and in *aggE* between 985 and 1,003), resulting in amplified fragments for *aggL* of 707 bp and for *aggE* of 875 bp (Figure 5). For the second deleted region, since it is located in a repeated region composed of sequences completely identical to each other, it was not possible to design primers with a unique position. The results of PCR amplification support the conclusion that this region is variable, not only between lactococci and enterococci, but also among enterococci, or is still under stabilizing selection (Figure 6). A comparison of the ability of aggregation and the size of the deleted region in *aggE* gene/protein indicates that there is no corelation between the size of deletion and agregation, collagen, fibronectin and mucin binding ability, and biofilm formation. It seems that the structure and the presence of the respective domains, not the length of the peptide, is responsible for the expression of certain auto-aggregation phenotypes or adhesion ability, as demonstrated previously for AggLb (Miljkovic et al., 2016).

### The role of AggE in auto-aggregation, adhesion to components of ECM, and biofilm formation

The auto-aggregation ability of the wild-type strain BGGO9-28, derivatives of which carry *aggE* gene (BGZLS10-27/pAggEPX48 and MG7284/pAggEPX48) and their non-aggregating derivative carrying an empty plasmid (BGZLS10-27/pAZIL and MG7284/pAZIL) was measured for a period of 5 h and the results are presented in Figure 3B.

**Figure 7:**
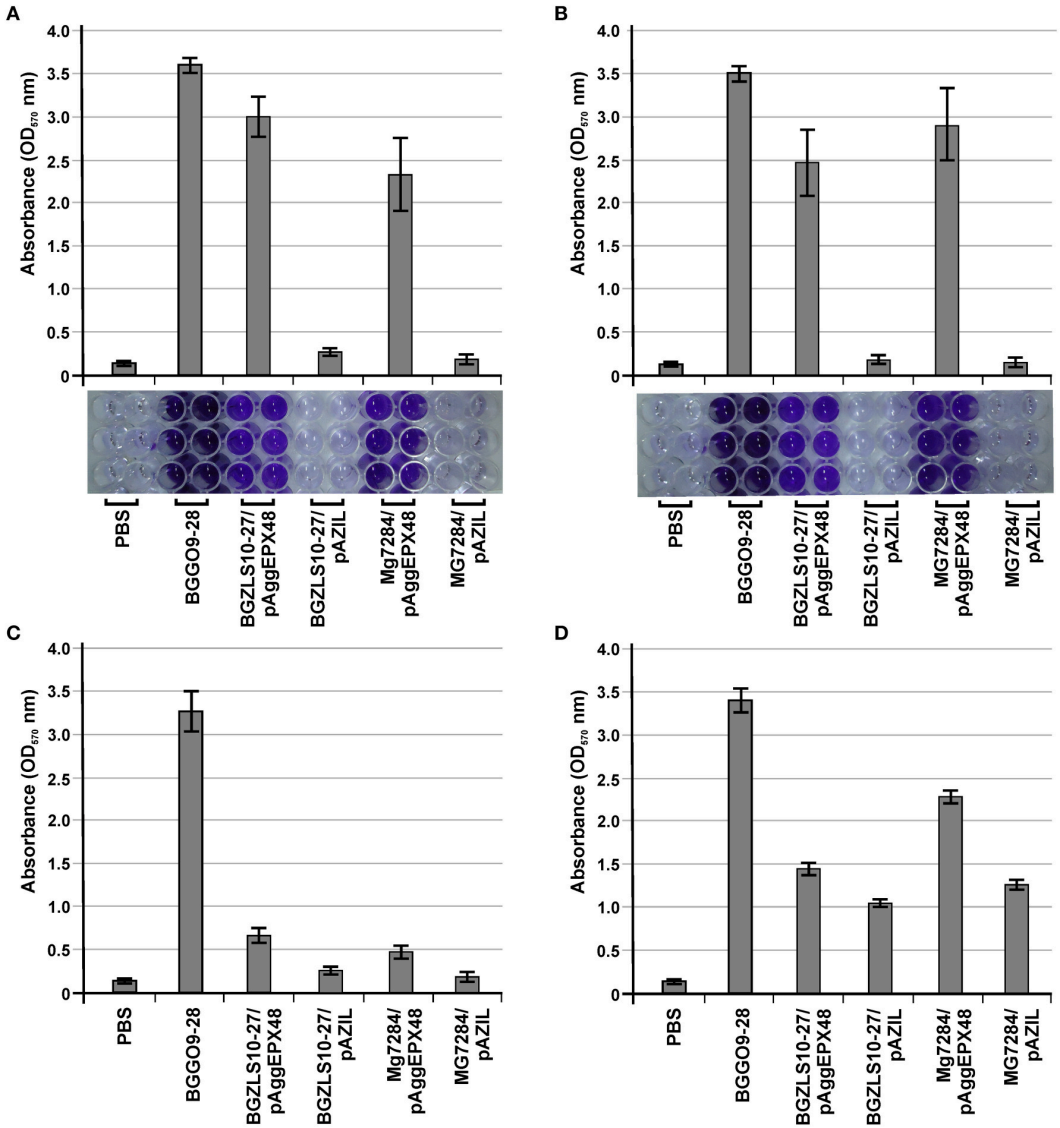
The role of AggE in adhesion to components of ECM and biofilm formation. Graphical presentation and photo of microtiter plates of results obtained in **(A)** collagen-binding assay; **(B)** fibronectin-binding assay; Graphical presentation of results obtained in **(C)** mucin binding assay and **(D)** biofilm formation assay. Results were expressed as average of normalized A_570_ values. The error bars show the standard deviations of three independent observations.

Earlier it was shown that isolates carrying *aggL* (the aggregation promoting factors from lactococci, AggL) or *aggLb* gene (the aggregation promoting factors from lactobacilli, AggLb) exhibited a direct correlation between auto-aggregation and their collagen binding ability, we tested the binding abilities of derivatives which carry *aggE* gene (BGZLS10-27/pAggEPX48 and MG7284/pAggEPX48) and their non-aggregating derivatives (BGZLS10-27/pAZIL and MG7284/pAZIL) to collagen. The different extents of adhesion to immobilized collagen (Figure 7A) and fibronectin (Figure 7B) were observed. BGGO9-28 exhibited the highest adhesion ability (absorbance value 3.54 to collagen and 3.48 to fibronectin), while the derivatives MG7284/pAggEPX48 (absorbance value 2.32 to collagen and 2.95 to fibronectin) and BGZLS10-27/pAggEPX48 (absorbance value 2.95 to collagen and 2.47 to fibronectin) possess slightly lower adhesion ability. Significant differences in the adherence to immobilized collagen and fibronectin were apparent between the mentioned aggregation-positive strains (BGGO9-28, MG7284/pAggEPX48, and BGZLS10-27/pAggEPX48) and their aggregation-negative controls, which showed almost no ability to bind to collagen or fibronectin (MG7284/pAZIL and BGZLS10-27/pAZIL). These results indicate a role for AggE in the specific interaction of BGGO9-28 with collagen and fibronectin on the cell surface. The higher adhesion ability of strain BGGO9-28 compared to the transformants indicates that some other factors in addition to AggE, present on the cell surface of the strain, are involved in the binding interaction to collagen and fibronectin.

Strain BGGO9-28 possesses a very high adhesion to mucin, while derivatives carrying *aggE* gene (BGZLS10-27/pAggEPX48 and MG7284/pAggEPX48) and their non-aggregating derivatives (BGZLS10-27/pAZIL and MG7284/pAZIL) showed lower adhesion abilities to mucin, with a small difference between them (clones showed only about two times higher binding ability) in contrast to binding to collagen and fibronectin (Figure 7C). It can be assumed that a direct correlation between the presence of the *aggE* gene and binding ability to mucin has not been determined (since AggE gives little contribution to mucin binding), confirming the specificity of the AggE binding factor for only certain components of the ECM. Interaction between bacterial cells is a complex process that involves a number of factors. It seems that for some processes such as auto-aggregation the most important is *aggE*/AggE, but other cell surface properties or environmental factors can contribute to the modulation of the expression of a given phenotype.

Evaluation of the formation of biofilms and their interaction with the bacterial surface molecules is a complementary approach to better understanding the mechanisms by which bacteria colonize a niche as a consequence of adaptation to environmental stresses (Peter et al., 2013). Aggregation and biofilm formation are multicellular processes that allow a community to be more resistant to stress conditions. Given that these are similar processes, it is not surprising that the same protein could be involved in both functions (Miljkovic et al., 2016). It is considered that the expressed *aggE* gene contributes to the increase of biofilm formation in a heterologous host, but it is not entirely (clones carrying AggE increased only about two times formation of biofim), indicated that additional factors/genes in enterococci are responsible for the full expression of this phenotype (Figure 7D).

### Probiotic characteristics of BGGO9-28 strain

The susceptibility of dairy isolate *En. faecium* BGGO9-28 to antibiotics according to CLSI (2015), and the presence of putative virulence genes, were determined in this study in the interest of safety considerations. Strain BGGO9-28 is sensitive to all of the antibiotics tested, without hemolytic or gelatinase activity, and does not carry any of the tested virulence determinants. Our results are in accordance with data that report the absence of these virulence factors in food-isolated *En. faecium* strains (Zheng et al., 2015); although previously, several starter and food-originated enterococci were shown to harbor some of the virulence factors, suggesting the importance of strain-specific properties for carrying certain virulence factors (Eaton and Gasson, 2001).

**Figure 8:**
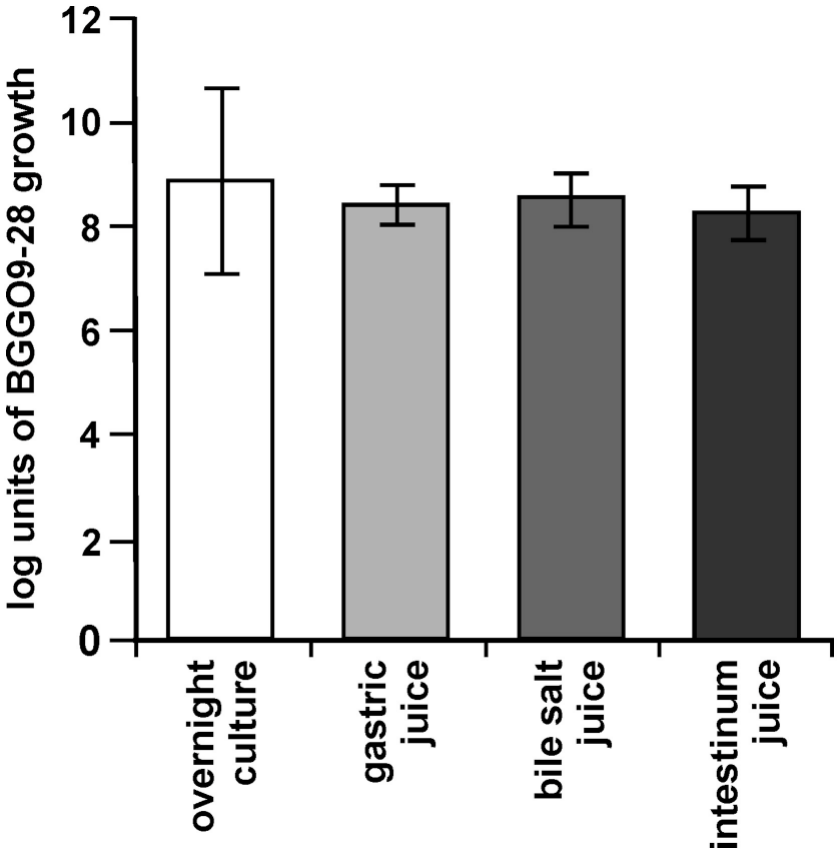
Survival of BGGO9-28 strain in GI tract conditions. Bacterial counts in suspensions made in 10% skimmed-milk of the *En. faecium* BGGO9-28 after chemically simulating the GI tract: in gastric, bile salt and intestinal juices. The error bars show the standard deviations of three independent observations.

The main desirable characteristic required for probiotic bacteria is their ability to survive during GI tract passage, in which low pH, bile salts, and digestion conditions are the main factors affecting this ability (Tan et al., 2013). The cells of strain BGGO9-28 were strongly maintained, with 8.30 log units in artificial gastric juice, 8.39 log units in bile salt juice and 8.22 log units for artificial intestine juice, respectively (Figure 8). It has been revealed that *En. faecium* BGGO9-28 was stable in acidic conditions (pH 2.0 for 3 h) and high bile salt, and had a high level of tolerance to pancreatin. BGGO9-28 has a high survival ability in the GI tract, which is similar to previous observations (Nueno-Palop and Narbad, 2011) reporting high levels of survival for enterococci.

Like the ability to survive under harsh conditions, adhesion is also an important functional characteristic of probiotic strains. The adhesion ability of *En. faecium* BGGO9-28 to the epithelial intestinal cell line Caco-2 was determined in order to define a percentage of attachment of the potential probiotic strain. The results obtained in this study clearly indicate that the strain possesses high adhesion abilities, about 80% adhesion to the Caco-2 cell line (data not shown).

The capability of probiotic strains to neutralize the negative effects of pathogens is their important health promoting property (FAO-WHO, 2006). One of the mechanisms of action is antimicrobial activity related to the production of bacteriocins, and colonization competition, or pathogen exclusion (Gareau et al., 2010; Satish-Kumar et al., 2011). Since strain BGGO9-28 does not exhibit antimicrobial activity, it has been proposed that the cell surface components involved in desirable probiotic features could be cell surface associated proteins, S-layer macromolecules built from proteins (Zhang et al., 2010) and auto-aggregation factors (Miljkovic et al., 2015).

It is important to emphasize that *in vitro* tests have many limits, because adhesion properties and mucus production depend on the cell line used in the study (Turpin et al., 2012). Additionally, some *in vitro* studies that estimated the degree of adhesion also indicate the cytotoxicity of tested strains to the target cells (Miquel et al., 2015). This suggests that adhesion and/or long-term colonization of probiotic agents usually is not without consequences.

This paper describes a new aggregation factor in enterococci, AggE, which belongs structurally to those of the other LAB, responsible for auto-aggregation and binding of carrier cells to specific components of the ECM. In conclusion, our findings indicate that there is a relationship between the auto-aggregation and adhesiveness of BGGO9-28 that is mediated by AggE on the cell surface. In particular, it should be noted that despite the fact that the adhesion of enterococci is generally extremely high, specificity of interaction with certain components of ECM is determined by AggE protein. The results of this study emphasize the direct link between AggE protein and auto-aggregation and binding to components of ECM, but also note the importance of other host and environmental factors on the overall adhesion of the bacterial cells.

## Author contributions

MK conceived, designed and coordinated this study, interpreted all of results and wrote this paper. KV, NP, and MM designed, performed, analyzed the experiments and wrote this paper. MT and AT provided experimental assistance, contributed to the preparation of the figures, provided technical assistance and contributed to the preparation of this paper. All authors reviewed the results and approved the final version of the manuscript.

### Conflict of interest statement

The authors declare that the research was conducted in the absence of any commercial or financial relationships that could be construed as a potential conflict of interest.
